# University of Nashville

**Published:** 1852-07

**Authors:** 


### UNIVERSITY OF NASHVILLE.

We have before us the Second Annual Announcement of the Medical Department of the University of Nashville, from which it appears that the class in that institution last winter, numbered one hundred and twenty-one. The number of graduates was thirty-three No medical school in our country ever had so prosperous a beginning.

